# Medication non-adherence in depression: a systematic review and metanalysis

**DOI:** 10.47626/2237-6089-2023-0680

**Published:** 2025-05-26

**Authors:** Roseane Dorte Halkjaer-Lassen, Walter S. Gonçalves, Bruno R. Gherman, Evandro S. F. Coutinho, Antonio E. Nardi, Maria A. A. Peres, José Carlos Appolinario

**Affiliations:** 1 Universidade Federal do Rio de Janeiro Instituto de Psiquiatria Ambulatório de Depressão Resistente ao Tratamento Rio de Janeiro RJ Brazil Ambulatório de Depressão Resistente ao Tratamento, Instituto de Psiquiatria, Universidade Federal do Rio de Janeiro (UFRJ), Rio de Janeiro, RJ, Brazil.; 2 Universidade do Estado do Rio de Janeiro Instituto de Medicina Social Departamento de Epidemiologia Rio de Janeiro RJ Brazil Departamento de Epidemiologia, Instituto de Medicina Social, Universidade do Estado do Rio de Janeiro, Rio de Janeiro, RJ, Brazil.; 3 UFRJ Escola de Enfermagem Anna Nery Rio de Janeiro RJ Brazil Escola de Enfermagem Anna Nery, UFRJ, Rio de Janeiro, RJ, Brazil.

**Keywords:** Compliance, adherence, major depressive disorder, antidepressant

## Abstract

**Objective:**

Medication non-adherence is frequently reported in patients with major depressive disorder (MDD). The objective of this review is to consolidate data on the prevalence of non-adherence to antidepressant in MDD.

**Methods:**

A systematic review with meta-analysis was performed according to the Preferred Reporting Items for Systematic Reviews and Meta-Analyses (PRISMA) guideline and the protocol was registered in International Prospective Register of Systematic Reviews (PROSPERO) under the number CRD42021199987. Studies assessing medication adherence in MDD were searched in PubMed/MEDLINE, Embase, Cumulative Index to Nursing and Allied Health Literature (CINAHL), and PsycINFO. The data extraction was performed by two independents authors. Meta-analysis used random effects model and performed a subgroup analysis.

**Results:**

From the articles retrieved, 11 studies were considered eligible for the final analysis. Most of them assessed non-adherence by self-report scales, followed by Pharmacy Dispensation Records, Monitoring Events Medication System (MEMS), and blood tests. The pooled proportion of non-adherence was 42% (95% confidence interval [95%CI] 30-54), but heterogeneity was very large (I² = 99%).

**Conclusion:**

Data from the selected studies suggests that a high number of individuals with MDD do not adequately take their medication as prescribed. The high heterogenicity of measures used for the assessment of adherence may have impacted the great variability of the results. The results suggest it is necessary that health care professionals should address this issue in order to achieve a better treatment outcome in major depression.

## Introduction

Depression, also known as major depressive disorder (MDD), is a common and serious medical condition that may cause acute and long-lasting symptoms of sadness or lack of interest in daily activities that usually interferes with individual's functionality. The treatment of depression is based on a multimodal approach that includes pharmacological and psychotherapeutic interventions.^1,2^ Overall, depression is considered a treatable mental disorder and the great majority of patients generally respond well to treatment, however approximately 30% of the patients with MDD did not respond adequately to the treatment.^3^ One of the most important issues related to the treatment of depression is patients’ poor adherence to antidepressant medications. Non-adherence to medications plays a crucial role in many cases of nonresponse, acute relapses, recurrences in the long term, and increased morbidity, comorbidity, and mortality.^4^

According to the World Health Organization (WHO), medication adherence can be defined as "the degree to which the person's behavior corresponds to the agreed recommendations of a health professional."^5^ Cramer et al.^6^ described adherence as an act of conforming to the recommendation made by the provider with respect timing, dosage, and frequency of medication intake. To estimate treatment adherence, some assessment tools were created, with their advantages and limitations. Scales, for example, are an easy to apply, have a low-cost, but are subject to interpretation bias, and have a high risk of inaccuracy. On the other hand, electronic devices have the advantage of recording the date and time when medication is taken, but some do not register which medication was taken, and is a more expensive method. In the case of evaluation by pharmacy registries, it has the easiness to record the drug dispensing of larger sample, but these methods are not able to assess whether the medication was taken in the correct dosages and times. At last, assessment of adherence using medication plasma levels is potentially the most accurate method, but has some limitation such as the high costs, it depends on a specific test for each antidepressant available, and it does not allow the evaluation of antidepressant levels in the long term, which can make it unfeasible to assess adherence in large samples.^6^ Even with this variety of adherence assessment methods, so far none has been considered the gold standard because the high possibilities of not express the real world.

The low degree of adherence creates obstacles in the treatment process, impairing the prognosis and resulting in negative consequences for the patients, such as high financial expenses and lowered quality of life.^7^ A growing number of evidences suggest that medication adherence in MDD is apparently low. Woo-Young et al. investigated the variations in discontinuation duration between different antidepressants in a real world treatment setting over a period of 6 months and found a discontinuation rate of 73%.^8^ Therefore, it is essential that patients recognize and accept their condition and understand the importance of following treatment correctly.^9^

In 2002, Pampallona et al.^10^ conducted the first systematic review of medication adherence in patients with depression. Among the studies included in this review, a mean adherence rate of 63% was found, but authors included in this review rates of adherence collect from antidepressant clinical trials and some studies that did not meet a standardized diagnostic criterion for depression.^10^ Another systematic review that was published in 2020 on the same topic found a 50% prevalence of non-adherence. However, this review also had the same limitations of the previous one.^11^

One of the major limitations of previous systematic reviews on this topic is the inclusion of studies involving participants hospitalized or participating in antidepressants clinical trials. Participants of those studies usually have their medication being monitored by a health care professionals and this could introduce an important bias in their results. Another important limitation was that these reviews included studies with participants with depressive symptoms and not only patients with MDD.

Considering the importance of an adequate medication adherence to improve depression outcomes and to overcome the limitations of previous reviews, the aim of this systematic review is to consolidate data on the prevalence non-adherence of antidepressant treatment in MDD.

## Methods

### Search strategy

This systematic review adheres to the Preferred Reporting Items for Systematic Reviews and Meta-analyses (PRISMA) guidelines and was registered in International Prospective Register of Systematic Reviews (PROSPERO) under the number CRD 42021199987. The electronic search was performed since database inception until March 2023 in the following databases: PubMed/MEDLINE, Embase, Cumulative Index to Nursing and Allied Health Literature (CINAHL), and PsycINFO. No filter for date of publication was applied. There were no language restrictions. We used the Medical Subjects Headings (MeSH): "depression," "antidepressant," "antidepressive," "adherence," "non-adherence," "dropout," "treatment refusal," "compliance," "discontinuation," and "persistence." The electronic search was complemented by a manual search for additional articles in reference lists and previous reviews to identify relevant publications that may have been missed (Supplementary Table S2).

### Inclusion and exclusion criteria

Only observational studies (cross-sectional studies and baseline data from longitudinal studies) were included. Reviews and systematic reviews were checked for identifying articles that were not retrieved in our electronic search. In addition, studies needed to meet the following criteria: (1) treatment adherence to antidepressant must be the primary outcome; (2) a validated method for measuring adherence to treatment should be used; (3) the studied samples must have a categorical diagnosis of depressive disorders (MDD) based on a stablished international classificatory system such as the Diagnostic and Statistical Manual of Mental Disorders (DSM) or the International Classification of Diseases (ICD). The exclusion criteria were case studies, case series, studies with samples that included children, adolescents or pregnancy woman, randomized controlled trials, and letters.

### Data collection and extraction

All articles were collected using Mendeley Reference Management software. Articles were organized into specific folders for each search database after the removal of duplicates. Two independent investigators (RDH-L and BRG) selected articles based on title and abstract according to the inclusion and exclusion criteria. Full article assessment was performed by these two authors. Unconformity was discussed and solved by consensus. In the absence of consensus, a senior author (JCA) was consulted. Data extraction was conducted by the first author (RDH-L) using an extraction data form designed for the purpose of this review. The data form included author, publication year, sociodemographic aspects, diagnosis, classification systems, assessment instruments for depression diagnosis, and non-adherence rates (percentage or crude values). Regarding longitudinal studies, only baseline data was collected.

### Quality assessment

The Newcastle Ottawa Scale (NOS) adapted for cross-sectional studies was used to assess the methodological quality of selected studies.^12^ Two authors (RDH-L and BRG) independently classified the studies with a "star system," ranging from 0-9 stars. The articles were judged in three dimensions: sample selection, comparability, and outcomes. This system allowed a semiquantitative evaluation of the quality of studies, being higher scores representative of better quality. Disagreements were discussed with the senior author (JCA) until there was a consensus.

### Statistical analysis – Meta-analysis

Heterogeneity was evaluated by inspecting the forest plot (point estimates and 95% confidence intervals [95%CI]) and the I² statistic. The I² can be interpreted as a measure of inconsistency across the findings of the studies.^13^ We used the random effects model to calculate the pooled proportion of non-adherence. A subgroup analysis was carried out to compare the studies that used validated instruments to assess adherence against those that applied other methods.

## Results

**Figure 1:**
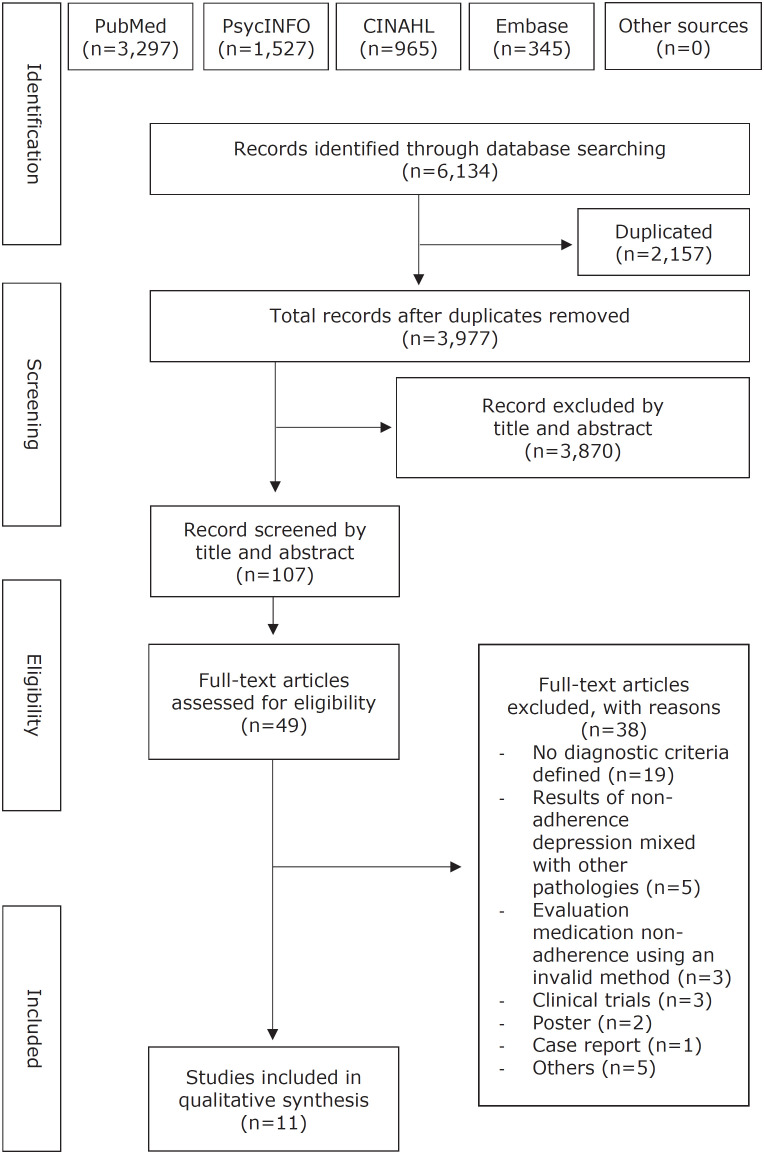
Flowchart illustrating study selection according to the Preferred Reporting Items for Systematic Review and Meta-Analyses (PRISMA). CINAHL = Cumulative Index to Nursing and Allied Health Literature.

This systematic review identified 3,977 articles after removal of duplicates. After the selection process by titles and abstracts, 49 studies were eligible for full text assessment. A total of 11 studies fulfilled criteria to be included in this systematic review (Figure 1). Overall, the included studies reported a non-adherence rate ranging from 14.7 to 70.3% (Table 1).

**Table 1:** Studies assessing prevalence of non-adherence to antidepressant medication in subjects with MDD

Author (country)	Study design	Sample (n)	Diagnostic system	Adherence assessment instrument	Definition of non-adherence	Non-adherence rate (%)
Bosworth^14^ (EUA)	Longitudinal	Mixed* (241)	DSM-IV	Morisky Green	0 point = adherence 1-4 point = non-adherence	28.0
Lu^15^ (China)	Cross sectional	Outpatient (135)	ICD-10	Morisky Green	0 point = adherence 1-4 point = non-adherence	62.2
Fawzi^16^ (Egypt)	Longitudinal	Outpatient (108)	ICD-10	GAM	N/A	43.6
Serrano^17^ (Spain)	Longitudinal	Outpatient (29)	DSM-IV	SMAQ	< 85% = non-adherence	27.6
Baeza-Velasco^18^ (France)	Cross sectional	Outpatient (360)	ICD-10	MARS	0-3 point = non-adherence	70.3
Chung-Hsuen^19^ (EUA)	Longitudinal	Outpatient (40873)	ICD-9	PDC	< 80% = non-adherence	49.8
Keyloun^20^ (EUA)	Longitudinal	Mixed* (527907)	ICD-9	PDC	< 80% = non-adherence	59.0
Rossom^21^ (EUA)	Cross sectional	Outpatient (177469)	ICD-9	Pharmacy Dispensation Records	Do not refill = non adherence	29.0
Freccero^22^ (Sweden)	Cross sectional	Population (8872)	ICD-10	Pharmacy Dispensation Records	Do not pick up medication = non-adherence	14.7
Roberson^24^ (EUA)	Longitudinal	Outpatient (56)	ICD-9	Blood sample	Undetected = non-adherence	29.0
Moon-Soo^25^ (Korea)	Longitudinal	Outpatient (76)	DSM-IV	MEMS	< 80% = non-adherence	47.4

DSM = Diagnostic and Statistical Manual; GAM = Global Adherence Measure; ICD = International Classification of Diseases and Related Health Problems; MARS = Medication Adherence Rating Scale; MEMS = Medication Events Monitoring System; N/A = not applicable; PDC = proportion of days covered; SMAQ = Simplified Medication Adherence Questionnaire.

* Outpatient and inpatient.

### Characteristics of the studies

The sample size of the studies varied from 29 to 527,907 participants, with a total of 756,169 participants in all 11 studies included. The mean age ranged from 31 to 69 years and the prevalence of females varied from 60 to 82.8%. In terms of assessment methods, five studies evaluated medication adherence by validated scales,^14-18^ four utilized pharmacy dispensing records,^19-22^ one used pill counts by Monitoring Events Medication System (MEMS),^23^ and one used medication levels in blood samples.^24^ Eight of the studies included outpatients,^15-19,21,23^ two included a mixed sample of outpatients and hospitalized patients,^14,20^ and one was performed in a populational sample.^22^ Concerning to study design, seven were longitudinal^14,16,17,19,20,24,25^ and four were cross-sectional^15,18,21,22^ According to the classification systems, three were based on DSM-IV,^17,20,25^ four on ICD-10,^15,16,18,22^ and four on ICD-9.^19-21,24^ Of note, most studies were performed in the United States.

### Studies assessing medication non-adherence through scales.

Five of the included studies employed validated scales and participants were categorized as adherent or non-adherent based on pre-defined cutoff points.

In 2008, Bosworth et al.^14^ examined the impact of antidepressant treatment adherence on MDD severity level of in 241 patients from a mixed sample. The medication adherence was evaluated by the Morisky Green Scale and the prevalence of non-adherence was 28%. The authors observed that non-medication adherence was a significant predictor for MDD severity.^14^ Using the same instrument, Lu et al.^15^ aimed to investigate the variables associated with adherence to antidepressant in 135 elderly Chinese outpatients with depression. The non-adherence rate of 62.2% was found and they highlighted that the participants with higher income had lower adherence rates.^15^

Baeza-Velasco et al.^18^ examined predictors of non-adherence in 360 outpatients with MDD who searched medical care due to a psychological decompensation. The evaluation of medication compliance was made through the Medication Adherence Classification Scale, a combination of the Morisky Medication Assessment Questionnaire (MMAQ) and the Medication Attitudes Inventory (MAI). The study found a prevalence of 70.3% of non-adherence. Psychiatric hospitalizations, suicidal ideation, medication side effects, and presence of physical pain were significantly higher in the non-adherence group.^19^

Fawzi et al.^16^ conducted a prospective study in 2012 to investigate the variables associated with medication adherence in 108 elderly patients with MDD. The evaluation of the adherence was made using the Global Adherence Measure (GAM). Based on the GAM scale, the authors found that 43.6% of subjects were non-adherent to their antidepressant regimen.^16^

At last, Serrano et al.^17^ evaluated medication adherence in 29 treated patients with MDD for 6 months from three primary care centers using the Simplified Medication Adherence Questionnaire Scale (SMAQ). They found a rate of 27.6% of non-adherence to the antidepressant treatment. The study also showed that participants who had high levels of medication adherence presented a higher reduction in depressive symptoms.^18^

### Studies that evaluated non-adherence through pharmacy dispensing records

Among the studies that evaluated medication adherence based on pharmacy dispensing records, two of these used a method called Proportional Covered Days (PCD). This method allows to estimated treatment adherence by calculating the proportion of days that the medication was available during the follow-up period. Observing if there was a delay in replacement of medication.^19,26^ In the other two studies, adherence was assessed by pharmacy dispensing control, considering adherent to treatment the patient who refilled their medication or requested a new prescription within the period estimated by the investigator.^21,22^

Chung-Hsuen et al.^19^ conducted a retrospective study using the MarketScan Commercial Claims and Encounters Database to assess the influence of the initial upward dose titration of antidepressant on medication adherence during the first 6 months of newly initiated treatments in patients with MDD. In this study, a total of 40,873 patients were divided into two groups: (1) those increasing the medication dosage (titration) and (2) those with a stable medication dose (non-titration) to evaluated medication adherence by the proportion of days covered (PDC). The authors found a percentage of patient non-adherence in both groups of 49.8%.^19^

Using the same methods, Keyloun et al.19 included data from 527,907 registered patients on a medical care database of insurance plans. To monitor the adherence rate over a year, they extracted records referring to the 3rd, 6th, 9th, and 12th months of treatment. The study found that non-adherence increased significantly over the course of a year, from 59% in the first evaluation at 3 months to 79% in the last evaluation at 12 months. It is important to mention that the authors pointed out that this rate of participation may not reflect reality, due to the incompleteness of records of this database.^20^

To assess factors associated with early medication non-adherence in the United States, Rossom et al.^21^ collected data from 177,469 adult patients from the Mental Health Research Network Data Consortium who had a new depressive episode and had to refill their prescriptions within a period of 180 days. Of these patients, 71% picked up their medication in the pharmacy and were considered adherent to antidepressant treatment. This study also noted that ethnicity may be a strong predictor for early non-adherence. Asian, non-Hispanic black, Hispanic, or native Hawaiian and Pacific Islander had an early non-adherence compared to non-Hispanic whites or Native American/Alaskan.^21^

Freccero et al.^22^ collected data from 8,872 patients with depression of the Primary Care Health Care Register in Sweden who were prescribed antidepressants. Among these patients, 14.7% were considered non-adherent because they had not picked up their first prescription within the period of 30 days. Among those who did not pick up their medication, 5.2% collected the prescriptions after 31 days, and 9.7% did not pick up the medication at any point in the study period. The study showed that elderly participants had a higher adherence compared to younger ones. Those born in the capital had a higher pick-up rate compared to those born in other cities, and married patients had a higher pick-up rate compared to patients with other marital status.^22^

### Studies evaluating non-adherence using a counting device

Only one study evaluated the medication adherence by a counting pills device called MEMS. The MEMS is a bottle that registers how many times subjects opened it to obtain their medication. Moon-Soo et al.^25^ conducted a study in 76 patients with MDD treated with antidepressant monotherapy in Korea. They reported a non-adherence of 47.4%.

### Studies that evaluated medication non-adherence through a blood sample

To assess treatment nonadherence, Roberson et al.^24^ used a discarded blood sample from 56 individuals who were treated with sertraline, citalopram, bupropion, or venlafaxine to evaluate the presence of these antidepressants in the bloodstream. Those patients who presented an undetectable level of their respective antidepressant, in the biomarker sample, were considered non-adherent. Overall, a rate of 29% non-adherence was reported for all antidepressants studied.^24^

### Quality assessment appraisal

According to the NOS, six longitudinal studies were classified as fair and only one was classified as good. All cross-sectional studies were considered fair. Lower scores were attributed to the category ascertainment of the exposure, and none of studies performed a sample size calculation (Supplementary Table S1).

### Meta analyses

**Figure 2:**
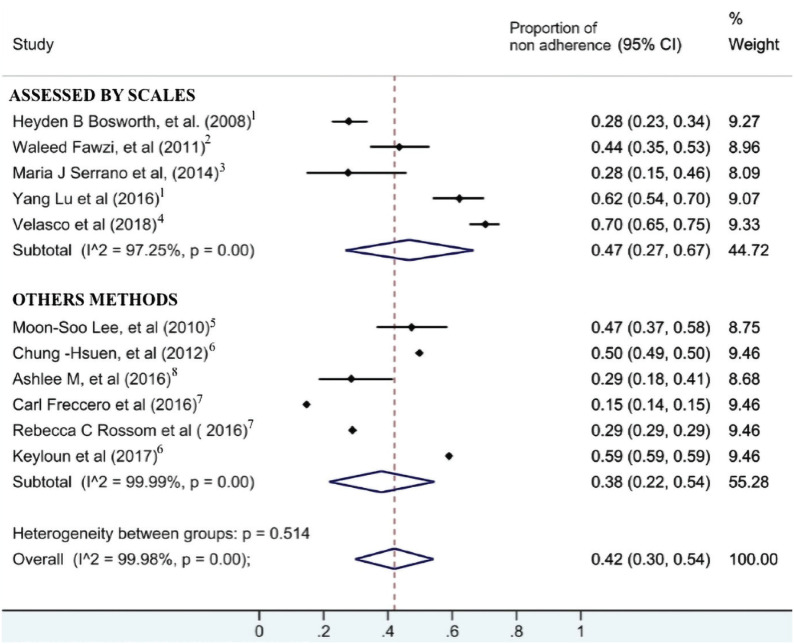
Forest plot. ^1^ Morisky Green Scale; ^2^ Global Adherence Measure (GAM); ^3^ Simplified Medication Adherence Questionnaire (SMAQ); ^4^ Medication Adherence Rating Scale (MARS); ^5^ Medication Events Monitoring System (MEMS); ^6^ Proportion of days covered (PDC); ^7^ Pharmacy Dispensation Records; ^8^ Blood sample.

The pooled proportion of non-adherence was 42%, but this finding must be considered cautiously due to the large heterogeneity between the estimates of the studies’ proportion. I² statistic was almost 100%, meaning that most of the observed variance was real. That is, the observed variance between studies cannot be explained by chance. When we stratified the studies considering the assessed by scales or by others instrument to evaluate adherence (subgroup analysis – see Figure 2), the heterogeneity was still large within each subgroup (I² > 97% in both subgroups). This means that the large variability of the study's findings was not consequent to this methodological difference.

## Discussion

Adherence to antidepressant medication is an important pillar of a successful treatment in MDD. This study updates the information of previews reviews regarding non-adherence to medication in individuals with MDD. We found 42% of non-adherence, but the huge heterogeneity requires caution when interpreting this finding. Besides the subgroup analysis that stratified the studies according to the use or not of instruments to assess adherence, the small number of studies and the missing information in some variables prevented us from going any further in exploring possible causes of heterogeneity.

The large variability in the results may be explained by the use of different definitions of medication adherence in the included studies, differences in the characteristics of the samples and instruments used to evaluate adherence to treatment. Table 1 summarizes the results obtained.

The high variability in the prevalence of medication adherence in depression was also reported in the review published in 2002 by Pampallona et al.^10^ They collected quantitative evidence on treatment adherence in depression and found a range of 3 to 30% of non-adherence in epidemiological studies. Furthermore, the most recent systematic review with meta-analysis on the theme was published by Semahegn et al.^11^ in 2020. In this review, the authors aimed to summarize factors associated with non-adherence to psychotropics in major psychiatric disorders.^11^ Of the 35 studies included, 16 assessed adherence in depression and included in total 42,255 participants with this condition. The authors reported a pooled prevalence of 50% (95%CI 40-59) of non-adherence in depression.

Both studies brought important data on the topic, emphasizing that non-adherence is still a challenge in the success of the treatment in patients with depression. Furthermore, Semahegn et al.^11^ highlighted several factors that may contribute to treatment success, such as unemployment, low education level, and age over 60 years old. However, these reviews had some methodological flaws that may have impacted their results, such as inclusion of studies that did not define specific diagnostic criteria of depression, language restriction, the use of non-validated instruments to assess adherence, and the inclusion of qualitative studies. Thus, we emphasize the need to standardize the measurement of medication adherence by validated instruments with a satisfactory level of accuracy, besides the importance of confirming the diagnosis of MDD.

Regarding the methodological quality of the studies included in this review, we observed that two assessment criteria items were decisive in compromising the quality of the studies: the absence of sample size calculation and the item on determining the exposure category. Calculating the sample size is essential for obtaining accurate prevalence estimates, in order to avoid findings that do not represent the real prevalence in the populations studied.

Some limitations should be considered in our study. First, although terms and strategies have been planned to cover the databases of literature in a comprehensive manner, the omission of relevant articles cannot be ruled out. The small number of studies and the large heterogeneity prevented us to evaluate the risk of publication bias. Secondly not using other data source may have impacted our results. However, to our knowledge, this review is the first to include only studies that presented a formal diagnosis of depression and that used validated adherence assessment methods. Our study followed the PRISMA guideline, and in addition, a quality assessment appraisal the selected articles was performed. Due to the high heterogeneity of the data found, such as sample type, methods of evaluation of medication adherence it was not feasible to synthesize the findings to estimate a precise answer to the research question.

Thus, we highlight the role of nurse as educators, as they have a positive influence on the treatment process and can help subjects to change their attitudes towards depression and improve their knowledge to increase their adherence treatment. Therefore, strategies have been developed to increase adherence, such as health education by telephone, assessment of adherence barriers, continuous monitoring of symptoms and side effects, providing feedback on treatment progress, among others.^27^

To be able to detect more accurately the prevalence of non-adherence in subjects with depression, future research should focus on standardizing methods to evaluate adherence to medication in order identify and understand the factors associated with non-adherence and, based on this, apply case-specific strategies to improve adherence.

## Conclusion

Unfortunately, the available studies adopt different methods for assessing adherence, which can result in discrepancies between the results. Nevertheless, this systematic review and meta-analysis found that medication non-adherence in subjects with MDD is still a current problem. It is urgent to develop strategies that encourage patients to take their medications correctly in order to increase the chance of getting the benefits from pharmacological treatments. However, even though this factor may influence our outcome, it is notorious that a considerable portion of patient with depression do not adequately adhere to treatment.
