# Supplementary material for: Medication non-adherence in depression: a systematic review and metanalysis
Source: Trends Psychiatry Psychother. 2025 May 26;47:e20230680. doi: 10.47626/2237-6089-2023-0680 (PMC12908919; doi:10.47626/2237-6089-2023-0680)
Supplement: Supplementary file 1 [file 2238-0019-trends-47-e20230680-Suppl01.pdf]

http://doi.org/10.47626/2237-6089-2023-0680

Supplementary Table S1 - Quality appraisal of the publications included in systematic review based on the NOS adapted for cross-sectional studies

		Selection			Outcome		Additional	Total
**Author (country)**	**Study design**	**Representativeness of the sample**	**Sample size**	**Ascertainment of the exposure (risk factor)**	**Assessment of the outcome**	**Statistical test**	**Confounders**	
Waleed Fawzi[15] (Egypt)	Cross sectional	*		*	*	*	*	5/9
Moon-Soo[24] (Korea)	Longitudinal	*		**	***	*	*	8/9
Chung-Hsuen[18] (EUA)	Longitudinal	*		*	**	*	*	6/9
Baeza-Velasco[17] (France)	Cross sectional	*		**	*	*	*	6/9
Bosworth[13] (EUA)	Longitudinal	*		**	*	*	*	6/9
Serrano[16] (Spain)	Longitudinal	*		**	*	*		5/9
Roberson[23] (EUA)	Longitudinal	*		*	**	*	*	6/9
Rossom[20] (EUA)	Cross sectional	*		*	**	*	*	6/9
Keyloun[19] (EUA)	Longitudinal	*		*	**	*	*	6/9
Freccero[21] (Sweden)	Cross sectional	*		*	**	*		5/9
Lu[14] (China)	Cross sectional	*		*	*	*	*	5/9

Newcastle-Ottawa Scale (NOS) total score = 9 points. No star = 0 point; * = 1 point, ** = 2 points.

Supplementary Table S2 - Search strategies for each database

	Database – Search terms			
	**PubMed**	**PsycINFO**	**CINAHL**	**Embase**
#1: (Patient/Problem)	(depression[mh] or depression[tiab] or antidepressant[mh] or antidepressant[tiab] or antidepressive[mh] or antidepressive[tiab])	“depression or “antidepressant” or “antidepressive”	“depression or “antidepressant” or “antidepressive”	“depression or “antidepressant” or “antidepressive”
#2: (Outcome)	(adherence[mh] or adherence[tiab] or non-adherence[mh] or non-adherence[tiab] or dropout[mh] or dropout[tiab] or treatment refusal[mh] or treatment refusal[tiab] or compliance[mh] or compliance[tiab] or discontinuation[mh] or discontinuation[tiab] or persistence[mh] or persistence[tiab])	“adherence” or “non-adherence” or “dropout” or “treatment refusal” or “compliance” or “discontinuation” or “persistence” or “compliance	“adherence” or “non-adherence” or “dropout” or “treatment refusal” or “compliance” or “discontinuation” or “persistence” or “compliance	“adherence” or “non-adherence” or “dropout” or “treatment refusal” or “compliance” or “discontinuation” or “persistence” or “compliance

Filter: until March 2023. CINAHL = Cumulative Index to Nursing and Allied Health Literature.
